# Availability of Volunteer-Led Home-Based Care System and Baseline Factors as Predictors of Clinical Outcomes in HIV-Infected Patients in Rural Zambia

**DOI:** 10.1371/journal.pone.0049564

**Published:** 2012-12-07

**Authors:** Christopher B. Estopinal, Janneke H. van Dijk, Stanley Sitali, Hannah Stewart, Mario A. Davidson, John Spurrier, Sten H. Vermund

**Affiliations:** 1 Vanderbilt University School of Medicine, Nashville, Tennessee, United States of America; 2 Macha Research Trust, Macha, Zambia; 3 Macha Mission Hospital, Macha, Zambia; University of New South Wales, Australia

## Abstract

**Background:**

We assessed the impact of home-based care (HBC) for HIV+ patients, comparing outcomes between two groups of Zambians receiving antiretroviral therapy (ART) who lived in villages with and without HBC teams.

**Methods:**

We conducted a retrospective cohort study using medical charts from Macha Mission Hospital, a hospital providing HIV care in Zambia's rural Southern Province. Date of birth, date of ART initiation, place of residence, sex, body mass index (BMI), CD4+ cell count, and hemoglobin (Hgb) were abstracted. Logistic regression was used to test our hypothesis that HBC was associated with treatment outcomes.

**Results:**

Of 655 patients, 523 (80%) were eligible and included in the study. There were 428 patients (82%) with favorable outcomes (alive and on ART) and 95 patients (18%) with unfavorable outcomes (died, lost to follow-up, or stopped treatment). A minority of the 523 eligible patients (n = 84, 16%) lived in villages with HBC available. Living in a village with HBC was not significantly associated with treatment outcomes; 80% of patients in a village with HBC had favorable outcomes, compared to 82% of patients in a village without HBC (P = 0.6 by χ^2^). In bivariable analysis, lower BMI (P<0.001), low CD4+ cell count (P = 0.02), low Hgb concentration (P = 0.02), and older age at ART initiation (P = 0.047) were associated with unfavorable outcomes. In multivariable analysis, low BMI remained associated with unfavorable outcomes (P<0.001).

**Conclusions:**

We did not find that living in a village with HBC available was associated with improved treatment outcomes. We speculate that the ART clinic's rigorous treatment preparation before ART initiation and continuous adherence counseling during ART create a motivated group of patients whose outcomes did not improve with additional HBC support. An alternative explanation is that the quality of the HBC program is suboptimal.

## Introduction

Home-based care (HBC) is used as an adjunct to or replacement for traditional clinic-based HIV care programs, especially where public health services are strained [1]–[5]. HBC has been associated with better antiretroviral therapy (ART) outcomes in Malawi and South Africa [2], [6], increased ART adherence [3], [7], and decreased stigma [8]. Tempering these promising results is a body of data regarding the limited effectiveness of some HBC programs [1]. More data are therefore needed from diverse HBC interventions in a variety of settings to document their impact on patient outcomes [5].

### Background

Zambia, a country of 12 million people in Southern Africa, had an adult HIV prevalence of 13.1–14.3% in 2009 [9], [10], giving it the 6^th^ highest adult prevalence in the world. Zambia is one of the most poverty-stricken nations in the world, ranking 150/169 in the 2010 Human Development Index (HDI), a multidimensional measure of poverty that considers life expectancy at birth, mean years of schooling, expected years of schooling, and gross national income per capita when assessing a country's developmental status [11]. While the world's average HDI has increased 41% since 1970, only three countries, the geographically contiguous nations of Zambia, Zimbabwe, and the Democratic Republic of the Congo, have seen their HDI decrease. Zambia experienced a 2.1% decline [12] attributed to the collapse of copper prices in 1980, the influx of refugees fleeing civil wars in neighboring countries, and the rampant HIV epidemic.

With support largely from the United States of America in the form of the President's Emergency Plan for AIDS Relief (PEPFAR), Zambia's major HIV care scale-up has yielded positive results; prevalence and mother to child transmission rates are dropping while the proportion of patients with advanced HIV receiving ART has increased from 1.7% in 2005 to 68% currently [9]. National policy makers and local healthcare providers alike are striving to provide effective care in a cost-effective and sustainable fashion and HBC is of special interest. We sought to evaluate the impact of one such program in rural Zambia by comparing outcomes of patients living in villages served by a HBC group with outcomes of patients living in villages not served by a HBC group.

## Methods

### Ethics Statement

The study was approved by the Vanderbilt University Institutional Review Board. Since data were collected from chart review and no specific identifiers were retained in the database, written informed consent was not obtained, with permission of the Vanderbilt Institutional Review Board. As a retrospective chart review, the study was considered a “program review” and thus exempt from Zambian Institutional Review Board oversight.

### Study Site

Macha Mission Hospital (MMH) is a 208-bed inpatient hospital in Zambia's rural Southern Province that began an ART-based HIV care program in 2005. The provincial HIV prevalence for men and women age 15–49 in 2007 was estimated to be 15% [13]. The hospital's catchment area is roughly 80 kilometers in diameter, with a population of approximately 160,000 people [14]. We conducted our retrospective cohort study in June–July 2008 when the HIV clinic at Macha Mission Hospital followed ≈3000 HIV-infected patients, 1500 of whom were receiving ART. Medications were provided through the Zambian government in conjunction with AIDS-Relief, a consortium of faith-based organizations supported by PEPFAR.

### Home-Based Care System

**Figure 1:**
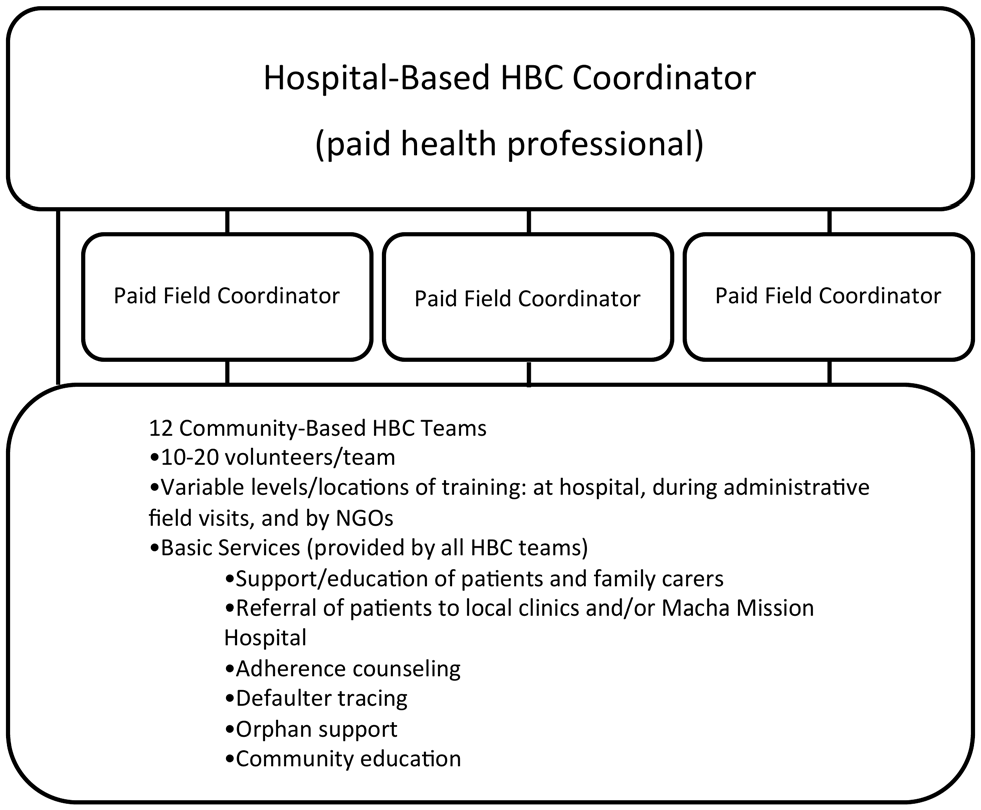
Home-based care (HBC) system organization.

The HBC program of the MMH is administrated by a clinical officer, who is responsible for resource allocation, volunteer training, and logistical support, and three paid field officers who train volunteers, monitor HBC activities, and provide site-level support for HBC groups. The care system is comprised of 206 volunteers distributed between 12 community-based HBC teams of 10–20 volunteer caregivers with variable levels of training obtained either through MMH, local nongovernmental organizations, or the Zambian government. The median distance between the HIV clinic and the HBC, as travelled by the shortest path accessible by motorcycle, is 14 km (IQR: 8.5–21.5, data available for 9 of 12 HBC groups; Dr. J Carlucci, unpublished data [14]. Each HBC team provides a heterogeneous panel of services and not every team provides the same services. At a minimum, most teams provide community education, referral of patients to local clinics or to MMH, adherence counseling, defaulter tracing, orphan support, and support and education of patients and family caregivers (Figure 1). Other services provided by some HBC teams include income-generating activities, nutritional support, psychosocial counseling, and basic hygiene support including bathing patients and house cleaning. HBC teams assist HIV-infected patients within a predetermined area around a central village; all HIV-positive patients who desire HBC services are enrolled in the program. HBC groups serve patients both receiving ART and not receiving ART. While caregivers work on a voluntary basis, some HBC groups are supported with uniforms, bicycles, and loans to be put toward income-generating activities. Records of specific services provided are not kept in a standardized format and a typically unavailable in the HBC program.

### Antiretroviral Initiation at the Macha Mission Hospital

In order to receive ART at the MMH clinic, a patient must first meet clinical criteria in the judgment of the clinician. Each patient's World Health Organization status [15] and CD4+ cell count are taken into account when making this decision. After being deemed eligible for ART per Zambian national guidelines, a patient must attend two intensive group counseling sessions, secure a “treatment buddy” who agrees to provide support to the patient, and maintain near-full adherence to a month-long co-trimoxazole and multivitamin regimen. Treatment is typically delayed if adherence goals are not met, although an individual clinician can initiate treatment if he or she believes immediate treatment is warranted. Most new patients receive tenofovir and emtricitabine with either efavirenz or nevirapine. Other patients, particularly those who have been in the program for a longer time, are treated with zidovudine or stavudine, lamivudine and lopinavir/ritonavir.

### Inclusion/Exclusion Criteria

All patients enrolled in ART from January 2006 to March 2007 were included in the study. Patients were excluded if they transferred out of care during the study period, they transferred into care during the study period, they were <18 years old at ART initiation, they received ART only for prevention of mother to child transmission (PMTCT), their charts were missing, or their charts were incomplete for village of residence, date of birth, date of ART initiation, or outcome. Patients who were missing body mass index (BMI), CD4+ cell count, or hemoglobin concentration (Hgb) were included. Baseline CD4+ cell count and Hgb were considered “missing” if values were not available within 100 days before ART initiation.

### Chart Review

Date of birth, date of ART initiation, place of residence, sex, BMI at ART initiation, CD4+ cell count at ART initiation, and Hgb at ART initiation were abstracted from patient medical charts. Age at ART initiation was determined using date of birth and date of ART initiation. Patients were classified as living in an HBC-serviced area if their village of residence was within the catchment area of one of the twelve MMH home-based care groups. BMI was calculated from heights measured at a patient's initial clinic visit and weights measured at a patient's ART initiation visit. The most recent CD4+ cell count and Hgb measured before or on the day of ART initiation were recorded.

**Figure 2:**
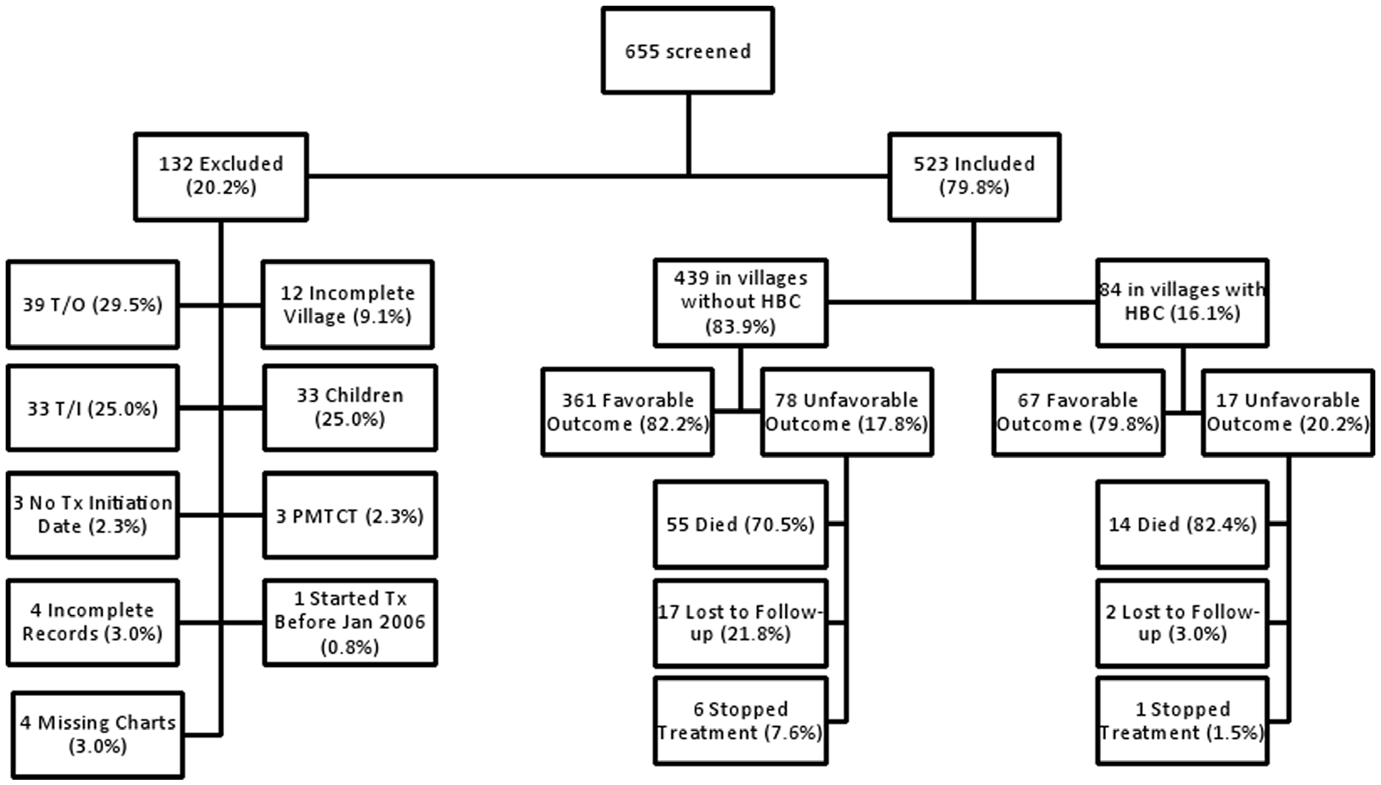
Study subject disposition (final study: n = 523). Patients were included or excluded based on criteria in the text. T/O: Transferred out of care. T/I: Transferred into care. No Tx Initiation Date: Patient has no recorded date of ART initiation. PMTCT: ART given only in peripartum period for prevention of mother to child transmission. Started Tx Before Jan 2006: Patient began receiving ART before January 1, 2006. LTFU: Lost to follow-up. Stopped Tx: Patient stopped taking ART.

Patient outcomes were assessed from medical chart reviews. Patients continuing to receive treatment at MMH until the time of the study were designated “alive and on ART.” Patients were classified as “dead” if they died after initiating ART. Patients were classified as “lost to follow-up” if they missed a clinic appointment without communicating a reason to the staff and were subsequently unable to be contacted over the ensuing 90 days. Patients were classified as “stopped treatment” if they voluntarily stopped taking ART. Patients were classified as “transferred in” if they initiated ART at another clinic and then began receiving ART at MMH. Patients were classified as “transferred out” if they stopped receiving treatment at the MMH HIV clinic but continued on ART at another clinic. Patients were classified as “PMTCT” if they only received ART perinatally. The outcome was defined as favorable (alive and on ART) or unfavorable (death, lost to follow-up, or stopped treatment). Patients in other outcome categories (transferred in, transferred out, and PMTCT) were excluded (Figure 2).

### Statistical Analysis

Data were managed using Excel™ (Microsoft Corp., Seattle, WA, USA) and analyzed using R: A Language and Environment for Statistical Computing (R Foundation for Statistical Computing, Vienna, Austria). Possible associations between outcomes and categorical variables were calculated using the Pearson χ^2^ test. The Wilcoxon rank sum test was used to assess associations between outcome and continuous variables. Multivariable logistic regression was used to determine the effects of variables on outcomes; all six independent variables (age at ART initiation, sex, BMI, CD4+ cell count, and HBC status) were included in the multivariable model.

## Results

### Subject Characteristics and Disposition

We reviewed the charts of 655 patients; 132 patients were excluded (20.2%), and 523 patients were included (79.9%) as per our study design. Of the excluded patients, 39 patients transferred out of care, 33 patients transferred into care, 33 patients were <18 years of age, 3 patients enrolled solely for PMTCT, 12 had incomplete village information, 3 had no date of ART initiation, 4 had incomplete outcome data, 4 had missing charts, and 1 started before January 2006 (Figure 2).

For BMI, CD4+ cell count, and/or Hgb data, 370 of 523 (70.7%) included patient charts were complete, 115 (22.0%) were missing one data point, 30 (5.7%) were missing two data points, and 8 (1.5%) were missing all three data points. Of the 523 included patient charts, 53 (10.1%) were missing BMI, 83 (15.9%) were missing CD4+ cell count, and 62 (11.9%) were missing Hgb. The demographics and patient characteristics of patients in areas serviced by an HBC group were well balanced when compared to patients in areas not serviced by an HBC group, with no statistically significant differences in any recorded variable between either group. (Table 1)

**Table 1:** Demographics and patient characteristics of patients living in communities with HBC available (HBC+) and not living in such communities (HBC−).

	HBC+ N = 84	HBC− N = 439	P Value
Age∧	30.8 35.3 43.5	31.9 38.2 45.7	P = 0.1*
Sex [female]	64%	62%	P = 0.6#
Body Mass Index [kg/m^2^]∧	16.3 18.3 20.2	16.5 18.5 20.4	P = 0.6*
CD4+ Cell Count [cells/µL]∧	77.0 139.0 204.0	86.5 146.0 198.0	P = 0.9*
Hemoglobin Concentration [g/dL]∧	10.0 11.0 12.0	9.0 11.0 12.0	P = 0.7*

∧ Value at ART initiation. a b c represent the lower quartile a, the median b, and the upper quartile c for continuous variables.

# Pearson Test.

* Wilcoxon Test.

### Outcomes

Of the 523 patients included in the study, 428 patients (81.9%) had a favorable outcome and 95 patients (18%) had an unfavorable outcome. In the unfavorable outcome group, 69 died, 19 were lost to follow-up, and 7 stopped treatment. Of 84 patients living in villages with HBC available, 80% had a favorable outcome, compared to 82% of the 439 patients living without HBC services (χ^2^ = 0.29, P = 0.6). Frequency of death, loss to follow-up, and stopping treatment rates were similar: 17%, 2%, and 1% respectively among patients living where HBC was available and 13%, 4%, and 1% among those living outside of HBC service areas. (Table 2)

**Table 2:** Outcomes of patients living in communities with HBC available (HBC+) and not living in such communities (HBC−).

	HBC+ N = 84	HBC− N = 439	P Value∧
Alive and on antiretroviral treatment	67 (80%)	361 (82%)	0.6
Died	14 (17%)	55 (13%)	
Lost to follow-up	2 (2%)	17 (4%)	
Stopped Treatment	1 (1%)	6 (1%)	

Percentages represent percentage of patients within HBC− or HBC+ group.

∧ P value compares Alive and on antiretroviral treatment (favorable outcome) to Died or Lost to follow-up or Stopped treatment (unfavorable outcome).

Bivariable analysis showed a significant difference in BMI, age, CD4+ cell count, and Hgb by outcome. BMI was higher in the favorable outcome group (P<0.001) with a median BMI of 18.7 kg/m^2^ (IQR (interquartile range): 16.8–20.6) in the favorable outcome group and a median BMI of 17.0 kg/m^2^ (IQR: 15.6–19.1) in the unfavorable outcome group. Age at ART initiation was greater in the favorable outcome group (F = 3.97, P = 0.047) with a median age of 38 years (IQR: 31.5–46.1) and a median age of 36 years in the unfavorable outcome group (IQR: 31.4–41.3). Hgb was higher in the favorable outcome group (F = 5.68, P = 0.02) with a median Hgb of 11 g/dL (IQR: 10–12) and a median Hgb of 10 g/dL in the unfavorable outcome group (IQR: 9–12). CD4+ cell count was also higher in the favorable outcome group (F = 5.76, P = 0.02) with a median CD4+ cell count of 151 cells/µL in the favorable outcome group (IQR: 87–203) and a median CD4+ cell count of 131 cells/µL in the unfavorable outcome group (IQR: 71–174). There were no statistically significant associations between outcome and patient gender. (Table 3)

**Table 3:** Outcomes of patients based on characteristics at ART initiation.

	Favorable Outcome& N = 428	Unfavorable Outcome∼ N = 95	P Value
Sex [female]∧	63% [n = 271]	56% [n = 53]	P = 0.2#
Age∧	31.6 38.2 46.1	31.4 36.1 41.3	P = 0.047*
Body Mass Index [kg/m^2^]∧	16.8 187. 20.1	15.6 17.0 19.1	P<0.001*
CD4+ cell Count [cells/µL]∧	87 151 203	71 131 174	P = 0.02*
Hemoglobin Concentration [g/dL]∧	10 11 12	9 10 12	P = 0.02*

& Favorable outcome is defined as “alive on antiretroviral therapy”.

∼ Unfavorable outcome is defined as “dead, lost to follow-up, or stopped antiretroviral therapy.”

∧ Value at ART initiation. a b c represent the lower quartile a, the median b, and the upper quartile c for continuous variables.

# Pearson Test.

* Wilcoxon Test.

Multivariable analysis showed no significant association between living in a HBC area and treatment outcome (P = 0.6). Lower BMI at the time of ART initiation was the only factor found to have a statistically significant association with unfavorable patient outcome in the multivariable analysis (P<0.001); as BMI decreased from 20.5 kg/m^2^ to 16.5 kg/m^2^, the odds of an unfavorable outcome more than doubled (CI: 1.4–3.2).

## Discussion

We did not find that availability of HBC services was associated with a favorable or unfavorable outcome in rural Zambia. Loss to follow-up was slightly less common in the HBC service communities, but the numbers were too small to derive inferences. Outcomes among all patients at the MMH HIV clinic were very good, possibly attributable to an intensive treatment preparation before ART initiation and to MHH's rigorous continuous adherence counseling program. This prepares all patients initiating ART for treatment success by offering ART only to those who have demonstrated adherence to followup appointments and non-ART medications and may diminish the measurable effects of HBC [14]. A favorable outcome rate of 81.9% is above that seen in other studies [16], [17]. Favorable outcome rates in the HBC-receiving groups in a published Malawi cohort were higher (96%) than ours (80%), while similar in those not receiving HBC (76% in Malawi vs. 82% in this study) [2].

Nonetheless, since the Malawi study showed favorable outcome rates comparatively higher in persons receiving HBC, we believe that additional factors are likely mitigating the effects of HBC on patient outcomes [2]. In Malawi, financial remuneration is provided in the HBC program, unlike in the MMH Zambia program described here. Programs staffed with volunteers who receive no compensation for services have been shown to have limited effectiveness and have difficulty retaining volunteers [18], [19]. Successful HBC programs typically provide either monetary or nonmonetary incentives for volunteers [20]–[22]. Malawian HBC workers received substantial incentives such as rain boots, raincoats, seed grain, and bicycles. These incentives were linked to volunteer activity and duration of commitment [2]. In contrast, while the MMH HBC groups were provided with uniforms, bicycles, and supplies, these were distributed sporadically and their distribution was not linked to either volunteer activities or duration of service.

Misclassification bias may have diluted the effect of HBC on patient outcomes. Patients were classified by HBC category based upon the village of residence listed in the medical chart. The spelling of many of these villages is variable – these villages have a single spoken name with several spellings. As our data were culled from written charts, variability in spelling may have led to misclassification of the exposure to HBC. This nondifferential misclassification of the exposure would bias results towards the null hypothesis, thus dampening any measured effect of HBC on outcomes [23]. Furthermore, we did not verify whether patients living in these areas received HBC services. This lack of verification may have also diluted our ability to assess the association between receipt of HBC services and patient outcome, and the results must therefore be interpreted with caution.

Finally, the effect of HBC in this population may not be detectable using our simple therapeutic outcome measure and/or with this sample size. HBC has been shown to impact patient quality of life in many ways not measured in this study, including but not limited to adherence [3], [7], health-related quality of life [4], increased acceptance and use of voluntary counseling and testing [4], [24], and decrease in HIV-related stigma [24]. The lack of outcome effect should therefore not be seen as labeling the HBC groups as ineffective but should rather suggest further research to characterize quality of life benefits possibly associated with receipt of HBC as well as the quantity and quality of services rendered.

Our data were consistent with the literature in terms of the association of unfavorable outcome with lower BMI, age, CD4+ cell count, and Hgb [25]–[28]. In multivariable analysis, lower BMI was the only factor that showed a statistically significant association with unfavorable outcome. Ongoing research in Zambia is pursuing early evidence that initiating low-BMI patients on ART may induce a physiologic phenomenon similar to the refeeding syndrome [29]. Our data strongly support the association between low BMI at ART initiation and unfavorable patient outcomes.

The goal of improving sustainability of PEPFAR-supported programs is essential, as current U.S. government financial support may wane over time and is already diversifying to include a broader range of global health care and prevention priority areas [30]–[32]. While HBC was not associated with a higher rate of positive patient outcomes in this study, outcome rates among both groups were favorable. Bolstering program organization and volunteer remuneration may strengthen the community health worker programs supported by PEPFAR.
